# Maternal psychological distress associates with alterations in resting‐state low‐frequency fluctuations and distal functional connectivity of the neonate medial prefrontal cortex

**DOI:** 10.1111/ejn.15882

**Published:** 2022-12-17

**Authors:** Olli Rajasilta, Suvi Häkkinen, Malin Björnsdotter, Noora M. Scheinin, Satu J. Lehtola, Jani Saunavaara, Riitta Parkkola, Tuire Lähdesmäki, Linnea Karlsson, Hasse Karlsson, Jetro J. Tuulari

**Affiliations:** ^1^ FinnBrain Birth Cohort Study, Turku Brain and Mind Center, Institute of Clinical Medicine University of Turku Turku Finland; ^2^ The Sahlgrenska University Hospital Gothenburg Sweden; ^3^ Department of Clinical Neuroscience Karolinska Institutet Stockholm Sweden; ^4^ Department of Psychiatry University of Turku and Turku University Hospital Turku Finland; ^5^ Department of Medical Physics University of Turku and Turku University Hospital Turku Finland; ^6^ Department of Radiology University of Turku and Turku University Hospital Turku Finland; ^7^ Department of Pediatric Neurology University of Turku and Turku University Hospital Turku Finland; ^8^ Center for Population Health Research University of Turku and Turku University Hospital Finland; ^9^ Department of Psychiatry University of Oxford (Sigrid Juselius Fellowship) Oxford UK; ^10^ Turku Collegium for Science and Medicine University of Turku Turku Finland; ^11^ Department of Paediatrics and Adolescent Medicine University of Turku and Turku University Hospital Turku Finland

**Keywords:** fALFF, neonate, prenatal stress, ReHo, rs‐fMRI

## Abstract

Prenatal stress exposure (PSE) has been observed to exert a programming effect on the developing infant brain, possibly with long‐lasting consequences on temperament, cognitive functions and the risk for developing psychiatric disorders. Several prior studies have revealed that PSE associates with alterations in neonate functional connectivity in the prefrontal regions and amygdala. In this study, we explored whether maternal psychological symptoms measured during the 24th gestational week had associations with neonate resting‐state network metrics. Twenty‐one neonates (nine female) underwent resting‐state fMRI scanning (mean gestation‐corrected age at scan 26.95 days) to assess fractional amplitude of low‐frequency fluctuation (fALFF) and regional homogeneity (ReHo). The ReHo/fALFF maps were used in multiple regression analysis to investigate whether maternal self‐reported anxiety and/or depressive symptoms associate with neonate functional brain features. Maternal psychological distress (composite score of depressive and anxiety symptoms) was positively associated with fALFF in the neonate medial prefrontal cortex (mPFC). Anxiety and depressive symptoms, assessed separately, exhibited similar but weaker associations. Post hoc seed‐based connectivity analyses further showed that distal connectivity of mPFC covaried with PSE. No associations were found between neonate ReHo and PSE. These results offer preliminary evidence that PSE may affect functional features of the developing brain during gestation.

**Abbreviations**
fALFF: fractional amplitude of low‐frequency fluctuations
mPFC: medial prefrontal cortex
rs‐fMRI: resting‐state functional magnetic resonance imaging

## 1 INTRODUCTION

Qualities of prenatal environment can have long‐term consequences for the developing individual (Van den Bergh et al., 2020). Recent cohort studies have revealed associations between exposure to prenatal maternal stress and subsequent developmental trajectories for the offspring, including effects on motor and cognitive development as well as increased risk for developing neuropsychiatric and affective disorders in vulnerable individuals (Van den Bergh et al., 2020). Moreover, prenatal stress exposure (PSE) has been linked to negative cognitive/emotional traits in infancy, for example, higher reactivity to external stimuli, and some effects may be different based on infant sex (van den Bergh et al., 2020). Emerging evidence also suggests that maternal pregnancy‐related anxiety (Buss et al., 2010), general and trait anxiety (Adamson et al., 2018) and depressive symptoms (van den Bergh et al., 2020) influence the developing architecture of the offspring brain.

Several elements have been proposed to underlie PSE induced adverse developmental trajectories, including dysregulation of the maternal hypothalamic–pituitary–adrenal axis (Van Bodegom et al., 2017), altered immunological and hormonal pathways (Merlot et al., 2008) as well as placental dysfunction (Bronson & Bale, 2016). Furthermore, growing interest has been channelled towards investigating gut‐brain interactions (Jašarević & Bale, 2019) and epigenetic DNA/histone modifications (Cao‐Lei et al., 2020) following PSE. Animal studies have observed that these factors associate with changes in brain monoaminergic signalling, axon and dendrite densities and gene expression (Fatima et al., 2017). Several of these cellular and molecular changes have been implicated as potential modifying factors in structural brain changes observed in in vitro studies (Dowell et al., 2019). On the other hand, while separate investigations into the causative mechanisms of PSE have led to important discoveries, it has become apparent that overall the mechanisms form a highly complex network of interactions, in which numerous detrimental and protective factors jointly determine future risks for pathology (Dufford et al., 2021).

As a counterpart for studies focusing on cellular level changes, neuroimaging studies have provided invaluable complementary information regarding in vivo macro‐scale brain development in the context of early exposures. Changes in brain structural architecture accompanying PSE have been documented in multiple age groups with consistent findings of alterations in grey matter volumes and cortical thinning in prefrontal, limbic, temporal and parietal regions (van den Bergh et al., 2018). However, less is known about potential changes occurring in the functional architecture of the brain even during normal development let alone following PSE. A common metric in resting‐state functional magnetic resonance imaging (rs‐fMRI) studies is functional connectivity (FC), which utilizes voxel‐wise spatiotemporal fluctuations of the BOLD‐signal to yield connectivity maps (Azeez & Biswal, 2017). Several rs‐fMRI studies have delineated how canonical functional brain networks develop over time from neonates' large and local into distributed and segregated networks seen in older children and adults (Gao et al., 2017; Gao, Alcauter, Smith, et al., 2015). Subtle alterations in static functional brain networks have been linked to several psychiatric disorders in adults (Takamura & Hanakawa, 2017) and also to variance in neonatal brain functions, e.g. emotional and language processing (Graham, Pfeifer, Fisher, Lin, et al., 2015).

Prior investigations into maternal psychological distress during the prenatal period have found functional and structural changes already present in the foetal and subsequently in the infant brain. Widespread changes in FC have been observed in foetal large‐scale brain networks with decreases and increases in FC strengths (de Asis‐Cruz et al., 2020). More specifically, functional networks governing more primal functions, i.e., somatosensory and salience functions, appear to exhibit stronger FC, whereas reduced FC are encountered in regions corresponding to executive control network and between inferior parietal lobule – occipital and fusiform gyri (de Asis‐Cruz et al., 2020). Moreover, a recent structural MRI study performed on foetuses revealed increased gyrification in frontal and temporal lobes, reduction in hippocampal volume with left‐lateralization, associating with maternal psychological distress (Wu et al., 2020). Regarding the neonatal and infant period, one study focusing on 6‐month‐old infants and maternal anxiety during pregnancy observed increased FC between the medial prefrontal cortex (mPFC) and amygdala; a key top – down control network governing emotionality, fear conditioning, anxiety and stress responses (Qiu, Anh, et al., 2015). Another study found an increased inverse relationship in FC between the amygdala and dorsal prefrontal cortex (dPFC) following exposure to prenatal maternal depression in 4 to 6 month old infants (Posner et al., 2016). Further, a post‐hoc analysis indicated that the observed connectivity changes were associated with an increase in foetal heart rate reactivity to mild maternal stressors (Posner et al., 2016), suggestive of increased reactivity to external stressful stimuli. A third study recorded decreased connectivity between brainstem, thalamus, insula, hypothalamus and the amygdala in association with PSE, as defined by a binary system regarding the presence of depression or anxiety in the maternal medical chart (Scheinost et al., 2016). However, this study was performed with preterm infants, which may limit generalizability. Fourth, a seed‐based FC report on neonates scanned at ~42 weeks postmenstrual age found that maternal IL‐6 and CRP levels during the 3rd trimester positively correlated with salience network FC with emphasis on the mPFC, temporoparietal junction and basal ganglia (Spann et al., 2018). This study focused on maternal immunological activation, which in turn has been linked with psychological distress. Finally, increased maternal perceived stress during the third trimester has been observed to associate with weaker neonate hippocampal‐cingulate cortex FC, which also subsequently correlated with infant memory performance at 4 months age (Scheinost et al., 2020).

Due to the prerequisite of an a priori region of interest, seed‐based FC measures alone are somewhat limited in revealing the nature of alterations across multiple brain regions and functional networks. However, these methods can uncover changes in network topology and connectivity strength. On the other hand, conserved networks commonly linked with later outcomes, such as the default mode network (DMN), may sustain their spatiotemporal features, decreasing seed‐based methods' sensitivity. To alleviate this problem, amplitude of low‐frequency fluctuations (ALFF) and fractional amplitude of low‐frequency fluctuations (fALFF) metrics have been developed and proposed to quantify low‐frequency fluctuation amplitudes of the BOLD‐signal (blood oxygen level dependent signal) (Zou et al., 2008). fALFF is typically defined as the power within the low‐frequency range (0.01–0.1 Hz) divided by the total power in the entire detectable frequency range, and represents the relative contribution of the low frequencies, thought to reflect spontaneous neural activity of the brain, relative to the whole frequency range (Zou et al., 2008). In healthy adults, high fALFF values are observed in regions that overlap with the DMN (Zou et al., 2008). In healthy neonates, high fALFF values are encountered within regions that govern primary functions, including sensorimotor and visual regions, whereas prefrontal areas exhibit lower fALFF values (Huang et al., 2020). fALFF has been shown to be a reliable method with moderate to high test–retest reliability and temporal stability (Küblböck et al., 2014; Zou et al., 2008), and sensitivity to pathological states in adults, in, for example, major depressive and bipolar disorders (Yang et al., 2019), cognitive impairment (Zeng et al., 2019) and schizophrenia (Xu et al., 2015). Regional homogeneity (ReHo) is another functional measure that has been demonstrated as an efficient, reliable and widely used index of local fMRI connectivity (Zang et al., 2004; Zuo & Xing, 2014). ReHo is formally computed using Kendall's correlation coefficient, and it is capable of measuring local connectivity changes with the premise that hemodynamic characteristics of each voxel in a functional cluster is often similar to the neighbouring voxels (Zang et al., 2004). In adults, ReHo shows promising sensitivity to local FC changes in several disease states, including schizophrenia (Liu et al., 2006) and cognitive impairment (Luo et al., 2018).

To the best of our knowledge, no previous studies have implemented fALFF and ReHo as primary metrics to investigate PSE associated changes in functional brain networks in neonates. We hypothesized that maternal psychological distress during pregnancy may inflict changes in neonate brain functional information processing with emphasis on regions within ‘vulnerable’ frontal networks that mature later in life, especially those involved in cognition, executive function, attention and emotional processing. Here, we sought to explore whether exposure to maternal self‐reported anxiety, depressive symptoms separately and their combined score, that is, psychological distress associates with changes in fALFF, ReHo and subsequent seed‐based connectivity maps in a sample of 21 neonates.

## 2 METHODS

This study was conducted in accordance with the Declaration of Helsinki, and it was approved by the Ethics Committee of the Hospital District of Southwest Finland (15.03.2011) §95, ETMK: 31/180/2011. Informed written consents were obtained from parents before MRI scans were conducted.

### 2.1 Participants

This study was performed as a part of the FinnBrain Birth Cohort Study (www.finnbrain.fi) (Karlsson et al., 2018). Twenty‐eight dyads of full‐term born healthy infants and mothers (Tables 1 and 2, respectively) were randomly recruited from the cohort to be included and participated in fMRI scans (scanned during year 2015). All mothers were healthy with no reported diagnoses of hypertension, hypercholesterolemia or diabetes mellitus. Exclusion criteria for infants included complications in neurological development, less than 5 points in the 5 min Apgar, previously diagnosed central nervous system anomaly, gestational age at delivery less than 32 weeks, and birth weight less than 1,500 g. All mothers reported having stopped ingesting alcohol and the use of illicit substances after being informed of being pregnant, but we included three participants with minor exposure to alcohol or illicit substances (marijuana) during very early gestation. The sample likely reflects the general population within the study catchment area (Yang et al., 2019). All scans were carried out during natural sleep at the gestation corrected age of 26.14 ± 6.28 days. To facilitate natural sleep, infants were fed with (breast) milk prior to the scanning session.

**TABLE 1:** Sample demographics of neonates (*N* = 21) included in the study

Variable	Whole sample (*N* = 21)	Boys (*N* = 12)	Girls (*N* = 9)
M ± SD (range)			
Age from birth (days)	26.95 ± 9.01 (11–53)	24.50 ± 7.67 (11–36)	30.22 ± 10.07 (23–53)
Age from term (days)	26.14 ± 6.28 (17–45)	23.17 ± 4.26 (17–30)	30.11 ± 6.23 (23–45)
Gestational age to imaging (weeks)	43.78 ± 0.91 (42.43–46.43)	43.39 ± 0.71 (42.43–44.43)	44.30 ± 0.93 (43.43–46.43)
Gestational age to birth (weeks)	39.93 ± 0.82 (38.14–41.43)	39.89 ± 0.85 (38.14–41.14)	39.98 ± 0.83 (38.86–41.43)
Offspring birth weight (grammes)	3524.76 ± 338.05 (3085–4395)	3562.50 ± 295.82 (3105–3980)	3474.33 ± 400.45 (3085–4395)
Offspring head circumference when born (cm)	35.29 ± 1.22 (33.0–37.5)	35.67 ± 1.21 (34.0–37.5)	34.78 ± 1.09 (33.0–37.0)
Apgar points at 1 min (MAD)	8.38 (1.15)	8.08 (1.22)	8.78 (0.25)
Apgar points at 5 min (MAD)	9.05 (0.46)	9.00 (0.49)	9.11 (0.40)
Frequencies			
Race/ethnicity (Caucasian/other)	21/0	12/0	9/0

*Note*: Variable selection was based on previous recommendations (Pulli et al., 2019).

Abbreviations: M, mean; SD, standard deviation; MAD, mean absolute deviation.

**TABLE 2:** Sample demographics of mothers (*N* = 21) included in the study

Variable	Whole sample (*N* = 21)	Boys (*N* = 12)	Girls (*N* = 9)
M ± SD (range)			
Age (years)	28.95 ± 4.20 (19–37)	29.08 ± 4.78 (19.00–37.00)	28.78 ± 3.56 (24.00–36.00)
Pre‐pregnancy BMI (kg/m^2^)	25.57 ± 4.05 (20.03–34.42)	25.92 ± 4.49 (20.03–34.42)	25.10 ± 3.59 (21.05–33.06)
EPDS	4.7 ± 4.2 (0–17)	3.8 ± 2.4 (0–8)	5.9 ± 5.7 (1–17)
SCL‐90	3.5 ± 3.2 (0–10)	3.8 ± 3.2 (0–10)	3.1 ± 3.3 (0–9)
Composite score	8.2 ± 6.6 (0–26)	7.7 ± 5.0 (0–17)	9.0 ± 8.6 (2–26)
Frequencies			
Maternal pre‐pregnancy BMI (kg/m^2^) (1 ≤ 25.00/2 = 25.00–29.99/3 ≥ 30)	10/7/4	6/3/3	4/4/1
Maternal monthly income (€) (1 ≤ 500 /2 = 501–1,000 /3 = 1,001–1,500/4 = 1,501–2,000/5 = 2,001–2,500/6 = 2,501–3,000/7 = 3,001–3,500/8 = 3,501–4,000/9 ≥ 4,000)	2/1/2/11/4/1/0/0/0	1/0/2/5/4/0/0/0/0	1/1/0/6/0/1/0/0/0
Maternal education level (1 = high school graduate or lower; 2 = college degree; 3 = university degree)	5/7/9	2/3/7	3/4/2
Maternal use of alcohol during pregnancy (yes/no)	3/18	2/10	1/8
Frequency of maternal use of alcohol during pregnancy (more than 1–2 times a month/1–2 times a month/less frequently)	0/1/2	0/1/1	0/0/1
Maternal use of illicit substances during pregnancy (yes/no)	1/20	0/12	1/8
Frequency of maternal use of illicit substances during pregnancy (more than 1–2 times a month /1–2 times a month/less frequently)	0/0/1	0/0/0	0/0/1

*Note*: Variable selection was based on previous recommendations (Pulli et al., 2019).

Abbreviations: M, mean; SD, standard deviation; EPDS, Edinburgh postnatal depression scale 10‐point questionnaire; SCL‐90, symptom checklist anxiety questionnaire.

### 2.2 Measures and procedures

Obstetric data was obtained from the Finnish Medical Birth Register of the National Institute for Health and Welfare (www.thl.fi) and included age from birth and term, gestational age when born, Apgar points at 1 and 5 min, birth weight, head circumference, maternal age in years, race/ethnicity, maternal pre‐pregnancy BMI, and exposure to alcohol and/or illicit substances.

All questionnaires assessing maternal psychological health were filled in by the mothers during the 24th gestational week. Maternal depressive symptoms during pregnancy were assessed by implementing the Edinburgh Postnatal Depression Scale (EPDS) (Cox et al., 1987), which has been validated for use during pregnancy. This 10‐item questionnaire is scaled from 0 to 30 points with a bigger score denoting increased symptom severity. Although no clinical cut‐off scores have been established, generally a score of 10 has been implicated as a clinically meaningful threshold for symptoms of depression in pregnancy (Vázquez & Míguez, 2019).

To assess maternal anxiety symptoms during pregnancy, the anxiety subscale of Symptom Checklist 90 (SCL‐90) was implemented (Holi et al., 1998). The SCL‐90 is widely used during the prenatal period (Lin et al., 2017; van den Heuvel et al., 2015). The SCL‐90 anxiety subscale consists of 10 items with a total score range of 0 to 40 with larger scores denoting increased symptom severity. As with EPDS, no clinical cut‐off scores have been established, but generally a score of 10 or higher has been evaluated to indicate clinically meaningful symptoms of anxiety (Karlsson et al., 2018; Korja et al., 2017). SCL‐90 and EPDS scores were combined to generate a measure of maternal psychological distress (henceforth referred to as the composite score). EPDS and SCL questionnaires have slightly different ranges, and to assure that directly summed scores provide a reliable composite measure, SCL‐90 and EPDS scores were standardized (mean = 0, SD = 1) and then summed. Non‐standardized and standardized sum scores correlated strongly (r_s_ = 0.992, *p* < 0.001).

### 2.3 Image acquisition

Altogether 28 infants underwent an MRI brain scanning session, including a 6‐min resting‐state fMRI sequence, conducted with a Siemens Magnetom Verio 3T MRI scanner (Siemens Medical Solutions, Erlangen, Germany) equipped with a 12‐element Head Matrix coil, allowing the use of Generalized Autocalibrating Partially Parallel Acquisition (GRAPPA) technique to accelerate acquisitions. Field‐of‐view (FOV) parameters were optimized for future replication by linear alignment to the anterior and posterior commissure lines. The total duration of the scanning protocol was 60 min, comprising of five major sequences in the following order: (1) Axial PD‐T2‐weighted TSE (Turbo Spin Echo), (2) Sagittal T1‐MPRAGE (Magnetization Prepared Rapid Acquisition Gradient Echo), (3) GRE field mapping, (4) DTI images and (5) a 6‐min duration EPI (Echo‐planar imaging) sequence. The rs‐fMRI sequence consisted of 42 slices with voxel size of 3.0 × 3.0 × 3.0 mm, TR of 2500 ms, TE of 30 ms, FOV of 216 × 216 mm and flip‐angle (FA) of 80°. Additionally, Fat Saturation Technique was used to reduce artefacts. Bandwidth was set to 1,310 Hz/Px for improved signal‐to‐noise ratio.

### 2.4 Image preprocessing

Data were slice timing corrected and motion corrected in FMRIB Software Library (FSL) v6.0 relative to a manually chosen reference volume, free of major artefacts. Motion outliers were estimated by ART (nitrc.org/projects/artifact_detect; motion composite < 2 mm, DVARS < 9). At this initial step, rs‐fMRI data of seven subjects were rejected from further analyses based on major artefacts (with most having more than 4/6 min of data outliers). Anatomical masks for white matter and CSF were defined by the UNC neonate atlas and registered to functional data with affine transformation using CONN toolbox (nitrc.org/projects/conn). Denoising consisted of nuisance regression with the first five principal components of the mean white matter and CSF signal and 24 motion covariates, linear detrending, and high‐pass filtering (0.008 Hz).

### 2.5 fALFF computation

The first outcome metric for neonate brain function was fractional amplitude of low‐frequency fluctuations (fALFF) that is estimated in a data‐driven manner and provides a voxel‐wise measure for BOLD‐signal amplitudes within frequency domains. *Z* values of fractional ALFF (fALFF) were used, based on prior work (Yan et al., 2013). Individual fALFF maps were computed with scripts from DPABI (rfmri.org/DPABI) for each subject (low frequency range 0.01–0.08 Hz). Preceding statistical group analysis, fALFF maps were normalized to the UNC neonate template (nitrc.org/projects/pediatricatlas) with 1.0 × 1.0 × 1.0 mm voxel dimensions. Finally, the data were smoothed with a Gaussian filter of 6 mm full width at half maximum (FWHM). Mean fALFF maps are provided in the supporting information.

### 2.6 ReHo computation

The second brain outcome metric was regional homogeneity (ReHo) that is also estimated in a data‐driven manner and generates a voxel‐wise local connectivity measure across the whole brain (Zang et al., 2004). ReHo is based on calculating the Kendall's coefficient of concordance over a target voxel and neighbouring voxels. ReHo was computed as implemented in DPABI (number of voxels in a cluster; *N* = 27) (rfmri.org/DPABI). Similarly, to the fALFF computation, ReHo maps were normalized to the UNC neonate template with 1.0 × 1.0 × 1.0 mm voxel dimensions. Data were smoothed with a Gaussian filter of 6 mm FWHM. For ReHo analysis, global outliers were included as nuisance covariates in regression and scrubbed, that is, removed from later analysis.

### 2.7 Seed‐based connectivity analysis computation

The seed‐based connectivity analysis (SCA) were performed with FSL tools with the use of identical preprocessing and nuisance regression as for the fALFF analyses (see above). Seed region‐of‐interest (ROI) was defined by a 3 mm radius sphere generated in FSL's (Jenkinson et al., 2012) FSLeyes, corresponding to the location (medial prefrontal cortex, mPFC) of the fALFF result in the UNC neonate template space. Seed‐based connectivity maps were then generated using FSL v6.0 fMRI Expert Analysis Tool (FEAT) (Woolrich et al., 2001). Seed ROIs were warped from template to subject space before extracting time series information. To obtain subject‐specific inverse transformations, FMRIB's Linear Image Registration Tool (FLIRT) (Jenkinson et al., 2002) was first applied with 12 degrees‐of‐freedom, referencing a sample specific fMRI template obtained by coregistering and averaging preprocessed fMRI data in the UNC neonate template space. Subsequently, FMRIB's Nonlinear Image Registration Tool (FNIRT) initialized by the affine matrix was used to estimate warps from subject to template space. These warping coefficients were further inverted by using FSL's ‘invwarp’ command and used to accurately transform the seed masks into the native space of each subject with the ‘applywarp’ command and nearest neighbour interpolation. Finally, average time series from seed ROIs were extracted using the ‘fslmeants’ command and a first‐level FEAT analysis was ran to assess the brain regions that had activity correlated to the mean mPFC activity. The resulting z‐score maps for each participant were then normalized to the UNC template space and group‐level statistical analyses were conducted in FSL's FEAT. Finally, the obtained group‐level SCA contrast maps were inclusively masked with the UNC neonate grey matter segmentation mask (probabilistic map thresholded at 0.5).

### 2.8 Statistical analysis

Statistical analyses were performed with SPM12 (fil.ion.ucl.ac.uk/spm/software/spm12/) and general linear models via ‘multiple regression’ design. The dependent variables were the fALFF and ReHo Z‐statistic maps in separate analyses. All models consisted of three independent variables (IV) of no interest that included neonate age at scanning session, sex and maternal pre‐pregnancy BMI (Rajasilta et al., 2021). Maternal adiposity, commonly measured with maternal pre‐pregnancy BMI, has been shown to associate with neonatal brain WM (Ou et al., 2015) and FC development (Li et al., 2016; Norr et al., 2021; Salzwedel et al., 2019; Spann et al., 2020), and thus, we included it as a covariate of no interest. For fALFF and ReHo statistical analyses, inclusive masking was performed with binarized UNC template GM mask (probabilistic grey matter map of the UNC atlas was thresholded at 0.8; performed with fslmaths) to limit effects within grey matter. For investigating maternal psychological distress, our primary model comprised of the composite score as the main explanatory variable (EV). As an attempt to distinguish whether this effect was more driven by mothers' subjective experiences of anxiety or depressive symptoms, multiple regression analyses were performed for SCL‐90 and EPDS scores separately. The statistical threshold was set at *p* < 0.001 and *p* < 0.005 (one‐tailed) for models investigating maternal psychological distress for ReHo and fALFF. All clusters that passed the initial uncorrected statistical threshold were further corrected with family‐wise error (FWE) correction at *p* < 0.001 and *p* < 0.005 at cluster level, respectively. For SCA, a more lenient threshold of *p* < 0.05 was used, with cluster‐level correction as above. To assert the validity of our underlying assumptions of the main parametric model, permutation‐based models were created for maternal psychological distress (supporting information) with identical designs by using Statistical non‐parametric mapping software (SnPM13; warwick.ac.uk/fac/sci/statistics/staff/acamedic‐research/nichols/software/snpm). Separate sensitivity analyses with additional covariates (neonate birth weight and maternal age in years) were performed to delineate possible interfering effects. The results of these analyses are presented in the supporting information. Further, an additional analysis excluding three of the neonates with mothers that reported minor illicit substances/alcohol during early pregnancy are presented in the supporting information. The brain areas were identified using the UNC‐neonate AAL atlas (Shi et al., 2011), and Mango version 4.0.1 (ric.uthscsa.edu/mango) and FSLeyes v0.34.2 were used for visualization.

## 3 RESULTS

**FIGURE 1:**
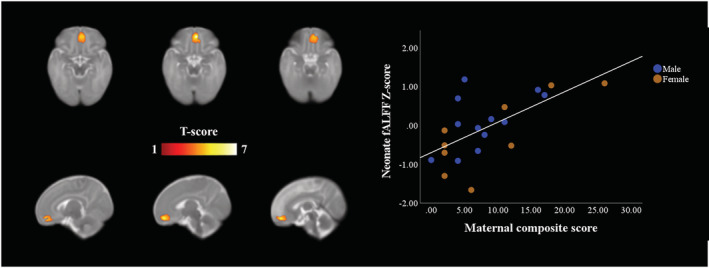
Regions where fALFF significantly correlated with maternal composite score (*p* < 0.001 FWE‐corrected) within the naturally sleeping neonate (*N* = 21). Highlighted region entails the ventromedial prefrontal cortex. Colour bar represents *T* scores. Images are displayed in radiological convention on the UNC neonate template on axial and sagittal slices. The graph on the right depicts the linear relationship between neonate vmPFC fALFF Z‐scores and maternal composite score. Abbreviations: A, anterior; P, posterior; L, left; R, right

**FIGURE 2:**
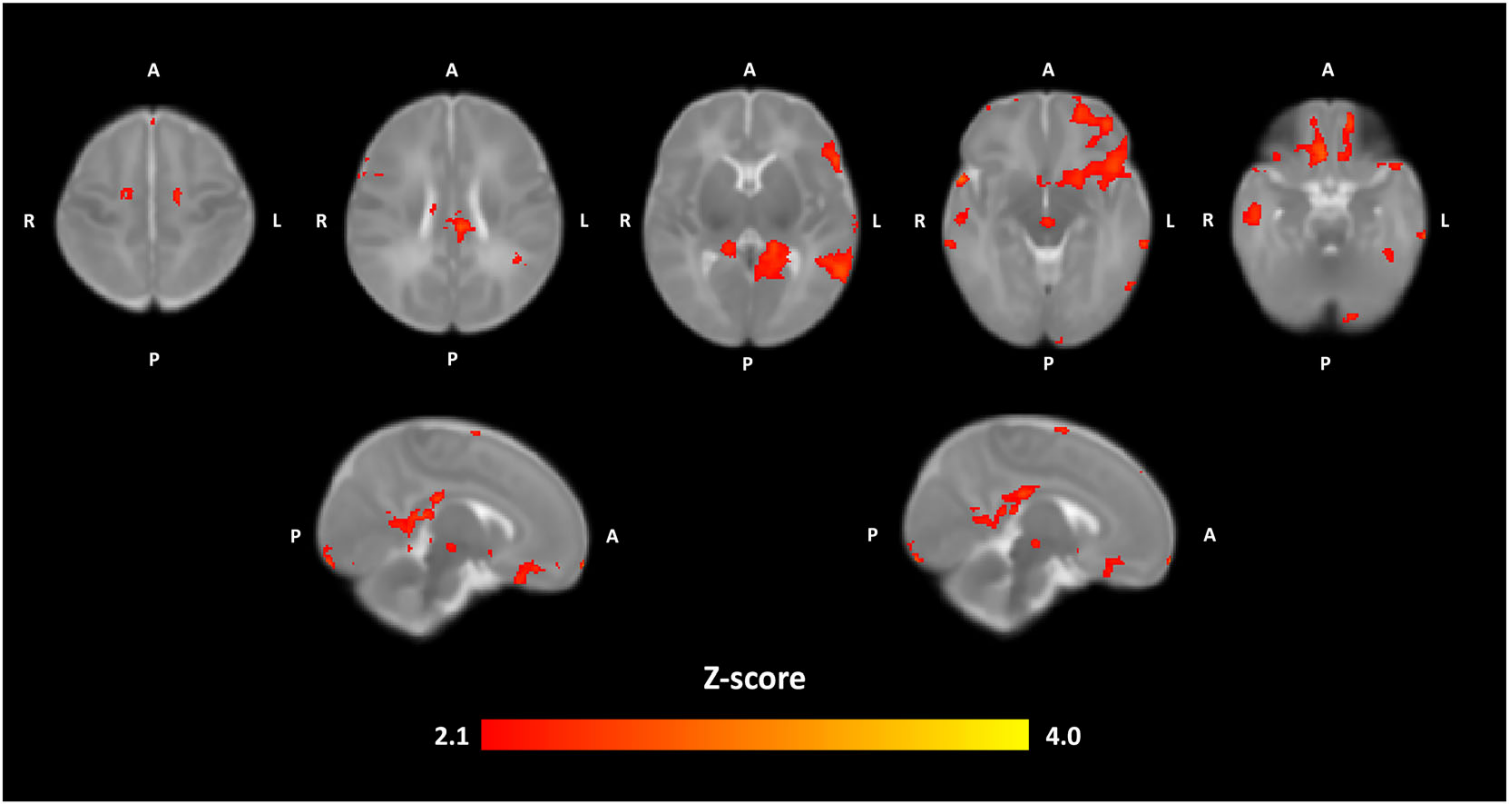
Brain regions where maternal composite score was positively correlated (Z‐score threshold of 2.1, corresponding to *p* < 0.05 multiple comparison corrected at cluster‐level) with neonate mPFC seed connectivity in multiple regression analysis (*N* = 21). The highlighted regions include the left orbitofrontal cortex, left calcarine cortex, middle and posterior cingulate cortices and the left middle temporal gyrus. MPFC seed ROI location and coordinates are displayed in Figure S4. Images are displayed in radiological convention on the UNC neonate template. Abbreviations: mPFC, medial prefrontal cortex; ROI, region of interest, A, anterior; P, posterior; R, right; L, left

In the main GLM model, a positive association was observed between the composite score and neonate fALFF in the medial prefrontal cortex (mPFC) (thresholded at *p* < 0.001, *p* < 0.001 FWE‐corrected *p* < 0.001, cluster size [kE] of 794 voxels). Effect size calculation yielded a large effect (ω^2^ = 0.547). The results are presented in Figure 1, and one sample *T*‐test results of the SCA are displayed in the Figure S4 to visualize the connectivity of the mPFC area that was implicated. Near‐identical results were obtained for SCL‐90 (*p* < 0.001 FWE‐corrected, kE 636) and EPDS scores (*p* < 0.004, FWE‐corrected, kE 482) when analysed separately, as compared to the original maternal psychological distress model. The only marked differences between these three results were in cluster size, that was largest when using the composite score and smallest when using only the EPDS score. No negative associations were detected between either maternal SCL‐90 score, EPDS score or the composite score and neonate fALFF maps. All peak cluster coordinates are provided in Table S2. The corresponding GLM analysis for ReHo found no statistically significant associations with maternal distress composite score, SCL‐90 or EPDS with statistical threshold of *p* < 0.001 or a more lenient threshold of *p* < 0.005. The post hoc SCA using the neonate mPFC as a seed region showed static FC divergence associated with PSE. Positive associations (*Z* score threshold 2.1, corresponding to *p* < 0.05, cluster‐corrected) were detected regarding PSE, between the mPFC seed region and left orbitofrontal regions, left calcarine cortex, left middle temporal gyrus, right inferior temporal gyrus and middle cingulate cortex (Figure 2). A small cluster was still observable with more stringent thresholding (*Z*‐score threshold 2.6, corresponding to *p* < 0.005, cluster‐corrected; see Figure S5). However, no clusters passing the statistical thresholding were observed with Z‐score threshold of 3.1, corresponding to cluster corrected *p* < 0.001. The calculated effect size yielded a large effect (ω^2^ = 0.151).

## 4 DISCUSSION

### 4.1 Summary of results

In this study, we investigated whether subjectively perceived maternal psychological distress (as quantified by the composite score and depression and anxiety scores separately) at the 24th gestational week associates with neonate fALFF and ReHo. Multiple regression analysis revealed a positive association between maternal composite score and neonate fALFF values in the mPFC. Corresponding positive fALFF associations were observed within the mPFC when using SCL‐90 and EPDS scores separately, albeit with smaller cluster sizes. We found no associations between neonate ReHo and PSE, suggesting that local static FC may not be altered. A post hoc SCA analysis with the neonate mPFC used as a seed region revealed several divergences in static FC with PSE. These results together suggest that PSE might not strongly influence static local functional connectivity but rather may influence distal static FC and other functional features of the neonate brain.

### 4.2 The possible underpinnings of fALFF and ReHo

Neonates exhibit high fALFF and ReHo in the sensorimotor, precuneus and cuneus regions (Huang et al., 2020) (see also Figure S1 for our data), suggesting that these regions have higher local synchrony and amplified resting‐state activity compared to other regions during early postnatal development. Several spatial divergences can be observed between these two metrics in this study: Increased fALFF values are also encountered in the temporal regions in a left‐lateralized manner, while increased ReHo values can be observed in the primary visual areas. Both metrics are increased in the basal ganglia region. These metrics differ fundamentally from each other in terms of measured domain; fALFF measures brain signal variability in the frequency domain, while ReHo measures signal similarity of neighbouring voxels in the time domain. In other terms, ReHo is an index of local FC (Zang et al., 2004), whereas fALFF reflects the intensity of resting‐state brain activity (Zou et al., 2008). Altered ReHo may associate with differences in distal FC (Rajasilta et al., 2021), likely reflecting intra‐ and inter‐network FC changes within and between groups of neurons.

As the ALFF and fALFF metrics measure amplitude and frequency change of the BOLD‐signal, their neurobiological interpretation is more challenging. The fALFF metric is credibly associated with neural activity (Aiello et al., 2015; Murta et al., 2015) and several other functional connectivity metrics (Sato et al., 2019), highlighting the possibility for overlapping alterations, e.g. changes in local and distal FC (Zhou, Shi, et al., 2019; Zhou, Tang, et al., 2019). Alterations, mostly increases, in fALFF values in a spectrum of pathological states (Du et al., 2019; Han et al., 2018; Zeng et al., 2019; Zhou, Tang, et al., 2019) suggest fairly good sensitivity but not necessarily neurobiological specificity. Indeed, abnormalities in cortical fALFF patterns can also be observed in subjects with white matter lesions, such as in Bell's palsy (Han et al., 2018), suggestive of reactive changes of neural firing patterns to altered whole network function. Furthermore, there are reports of distinct fALFF patterns associating with personality traits in healthy individuals (Ikeda et al., 2017; Wei et al., 2011) and mathematical disability in children (Jolles et al., 2016), advocating an interpretation for fALFF as a nonspecific marker of altered neural activity in a neural population. Therefore, fALFF may be sensitive for impaired neural function, but also for the reconfiguration of neural firing patterns. For example, a recent study found increased fALFF overlapping with network hyperconnectivity in children with mathematical disabilities (Jolles et al., 2016). These brain changes were further hypothesized to be related with disrupted network‐level flexibility, for example, the inability to coherently decrease and increase brain region activity when engaging in mathematical tasks (Jolles et al., 2016). Prior multimodal studies performed on adults have shown that fMRI and EEG signal amplitudes are modulated by effects of aging and sex (Zhong & Chen, 2020), with the hypothesis that as vasculature reactivity declines with age, so does BOLD‐signal amplitude (Zhong & Chen, 2020). Contrarily, frequency bands of MEG‐fMRI measurements follow similar power‐law distributions with electrophysiological signals, suggesting that intensity of low‐frequency modulations stem from neural activity rather than neurovascular coupling effects (Zhang et al., 2022). Finally, a recent multimodal study enrolling a small sample of male adults to be simultaneously scanned with PET, rs‐fMRI and EEG with focus on DMN demonstrated no correlations between rs‐fMRI ReHo and fALFF metrics, FDG‐PET and EEG microstates, but revealed positive correlations between rs‐fMRI degree‐centrality metric and EEG microstates (Rajkumar et al., 2021). EEG microstates however focus on simultaneous active sources, that is, whole‐brain networks, and may be insensitive to smaller functional alterations within networks (Michel & Koenig, 2018). To conclude, there is a void of knowledge regarding fALFF neural correlates and future multimodal EEG‐fMRI studies are required to disentangle electrophysiological and neurovascular mechanisms underlying frequency amplitude measurements in all age groups.

### 4.3 Maternal psychological distress associates with altered fALFF in the neonate mPFC

In the current study, PSE associated with increased fALFF within the neonate mPFC. In adults, the mPFC has robust structural and functional connectivity to the frontal lobe, cingulate cortices, temporal and parietal regions, amygdala and hippocampi, but also to the cerebellum and other subcortical nuclei (Alves et al., 2019). Parcellated into three distinct large regional domains, the ventral, anterior and dorsal medial prefrontal cortices (v/a/dmPFC, respectively), mPFC is involved in cognitive and affective functions (Lieberman et al., 2019). In the present fALFF analysis, the significant PSE effects were confined to the vmPFC, an area that is regarded as a part of a key hub for the DMN in neonates (Gao, Alcauter, Smith, et al., 2015) and adults (Raichle, 2015), and is strongly associated with emotion regulation, social cognition and value encoding in adults (Lieberman et al., 2019; Raichle, 2015). However, less is known about its roles, functional connectivity, and brain‐behaviour functions in neonates, especially across different states of wakefulness and sleep. Although neonates display a robust functional brain organization at birth (Rajasilta et al., 2020), the functional connectivity of these networks greatly differ from the mature networks in adults (Fitzgibbon et al., 2020), which is likely in part because of the fact that they are asleep (Horovitz et al., 2009). For example, adults scanned at rest have considerably higher fALFF values in the mPFC than neonates scanned during sleep (Huang et al., 2020). As functional brain networks undergo formidable changes during distinct sleep stages, consideration should be placed on the network in question and the topological changes that occur during sleep. In general, the amplitude of low‐frequency fluctuations increase during rest and early‐sleep stages in adults with region‐specific variability (Fukunaga et al., 2006). For example, in the adult DMN, frontal regions show functional decoupling from other key nodes within the network during sleep (Horovitz et al., 2009) and experience greater diurnal variations as measured by ALFF (Fafrowicz et al., 2019). Here, we show that maternal psychological distress amplifies the sleeping neonate fALFF in the mPFC, which may be interpreted as increasing the resemblance of mPFC's functional characteristics to that of an adult.

In adults, the vmPFC has robust structural and functional connectivity with the amygdala‐related brain circuit involved in internalizing disorders and fear conditioning (Kim et al., 2011), which may be of interest in future studies of neonates and infants, as PSE has been consistently associated with heightened emotional reactivity, fear and temperament phenotypes in neonates (Austin et al., 2005; Thomas et al., 2019) and young children (Blair et al., 2011). These behavioural outcomes have been further associated with reduced uncinate fasciculus integrity (Qiu et al., 2013)—a white matter tract that spans several key regions regarding emotionality, including the vmPFC and the amygdala. Comparably to the case of Bell's palsy discussed in two chapters above, where divergent fALFF patterns are observed following a white matter lesion, it is possible that the observed increased fALFF in mPFC in this study may point towards elevated neural efforts due to compromised WM tract integrity. Several prior reports have found links between maternal depressive or anxiety symptoms and neonate amygdala—vmPFC FC alterations (Qiu, Anh, et al., 2015; Thomas et al., 2019), suggesting that functional circuit is vulnerable for modifications by prenatal stress exposure.

Functional brain networks, especially those involved in higher order functions such as the DMN, emerge with diminutive distal connectivity in the neonate (Gao, Alcauter, Elton, et al., 2015; Gao, Alcauter, Smith, et al., 2015; Rajasilta et al., 2020) and follow a distinct maturation trajectory during which the topology of the network changes dramatically (Gao, Alcauter, Elton, et al., 2015; Gao, Alcauter, Smith, et al., 2015). It has been hypothesized that early life stress or adversities may accelerate functional network development by altering the connectivity between core regions (Gee et al., 2013; Herzberg et al., 2021; Tottenham, 2018), leading to more adult‐like network topology earlier in life. Moreover, preliminary data suggests that this process may be accompanied by changes in intra‐network and between‐network static FC in adulthood (Philip et al., 2013). Focusing on neonates at 3 weeks of age, we here showed that PSE modulates activity and connectivity of mPFC. The mPFC ROI showed stronger connectivity to the left orbitofrontal cortex, left calcarine cortex, middle and posterior cingulate cortices and left middle temporal gyrus. Not surprisingly, these changes in mPFC FC are concurrent with DMN developmental trajectory, albeit with lateralization to the left, as mPFC is considered a core hub in the network (Fransson et al., 2011; Gao et al., 2009; Gao, Alcauter, Smith, et al., 2015). Fitting prior models of DMN FC development, the FC changes in the DMN observed in this study resemble those that occur during the first year of life (Gao et al., 2009; Gao, Alcauter, Smith, et al., 2015). In this framework, given that the fALFF and ReHo have been reported to have similar regional patterns in neonates (Huang et al., 2020), it was slightly surprising that we found no concurrent changes in local static FC as investigated by our ReHo analysis across the whole brain. These findings together do however fit prior findings of reorganized network FC patterns with mostly increased distal FC after early‐life stress (Gao, Alcauter, Elton, et al., 2015; Graham, Pfeifer, Fisher, Carpenter, & Fair, 2015; Graham, Pfeifer, Fisher, Lin, et al., 2015; Philip et al., 2016), suggesting accelerated network development as a consequence of PSE. As the vmPFC has important FC with the limbic system and salience network, of which the latter is majorly involved in orchestrating shifting of functional brain states between intrinsic and extrinsic tasks, it may be of interest to future studies to explore whether alterations in FC and fALFF exerted during early life have implications for attentional processes later in life, when between‐network synchronization is consolidated. Prior studies investigating PSE effects on neonate brain network FC have obtained somewhat diverging results (Posner et al., 2016; Qiu, Anh, et al., 2015; Scheinost et al., 2016; Scheinost et al., 2020; Spann et al., 2018), with only two studies observing FC changes within the neonate mPFC (Qiu, Anh, et al., 2015; Spann et al., 2018). Differences in sample sizes, analysis methods as well as cohort demographics and factors such as scan timing after birth, assessments, severity and timing regarding PSE are likely to explain these incongruities. Future studies are required to assess the possibility that changes in fALFF could associate with alterations in dynamic functional states, possibly revealing novel mechanisms and changes following PSE.

Individual differences in epigenetic and genetic factors (O'Donnell et al., 2017; Qiu, Tuan, et al., 2015) are likely to play a role in determining repercussions of PSE on the development of functional brain architecture. While PSE and abnormalities in functional metrics have been commonly linked with adverse outcomes (Graham et al., 2019; Spann et al., 2018), less is known about PSE induced resilience factors and other possible positive outcomes. For example, transient severe stress during early‐mid gestation is associated with increased schizophrenia risk (Guo et al., 2019; Khashan et al., 2008), while continuous stress through the perinatal period associates with little psychiatric problems and increased markers for resilience (Serpeloni et al., 2019). To the best of our knowledge, no studies have implemented brain imaging techniques for PSE in search for resilience or protective factors for adverse outcomes. Several studies have however focused on early life stress and found that mild exposure is linked with resilience factors (Miller et al., 2020; Yamamoto et al., 2017) and even a buffer against accelerated biological aging (Miller et al., 2020) in the offspring. To conclude, although our results are in line with prior investigations that point towards alterations in the fronto‐limbic networks of the neonate brain following PSE, future studies are needed to assess whether and which of these observed changes are permanent and of negative or positive nature regarding infant outcomes. Moreover, extant literature and our results highlight the need for future research venues with directed focus on delineating protective and vulnerability factors that determine which individuals are at risk for developing adverse outcomes. Consistent findings could lead to promising strategies aiming to mitigate harmful stress exposure induced effects that might otherwise lead to adverse outcomes.

## 5 LIMITATIONS

Our small sample size, while common for functional neuroimaging research, may overestimate effect sizes and correlations due to sampling variability (Marek et al., 2022; Szucs & Ioannidis, 2020; Vul et al., 2009). Thus, even though we used stringent thresholds for statistical significance and confirmed that the results survive non‐parametric testing, we would like to caution that our results are preliminary and future studies with larger samples are required to validate our findings. Albeit performing a comprehensive motion clean‐up, we cannot rule out the possibility that the inclusion of some volumes with higher amounts of motion may influence the fALFF maps. Our cross‐sectional measurements do not allow causal inference, and the distress scales are based on self‐reports. Furthermore, there were only few subjects in our study with exposure to severe maternal psychological distress. Implementing more densely sampled questionnaires and objective measures, for example sleep data or cortisol/interleukin measurements would be of interest in future studies to further tease out the mechanisms behind the associations. Finally, the neurobiological basis of fALFF, ALFF and ReHo remain indeterminate and should be probed by future studies for example by simultaneous EEG/fMRI paradigms.

## 6 CONCLUSIONS

We report that maternal psychological distress during the 24th gestational week, as measured by the composite score of EPDS and SCL‐90 questionnaires, has a positive association with fALFF values in the neonate mPFC. We also found evidence of altered mPFC FC after PSE, consistent with the theoretical model of accelerated DMN development. No changes in static local FC were observed across the whole brain. Our results add to the cumulative evidence that exposure to maternal psychological distress during gestation may influence the developing functional brain of the neonate.

## CONFLICT OF INTEREST

The authors declare no conflict of interest.

## AUTHOR CONTRIBUTIONS

JJT, MB, NMS, LK, HK and MB planned and/or funded the MR measurements. JS planned and implemented the image acquisition parameters. JJT and SL collected the imaging data. OR and JJT planned the analytical approach and performed the data analyses. SH aided in the data preprocessing and interpretation. RP provided neuroradiological expertise for screening the acquired MRI images for incidental findings. HK and LK established the cohort and built the infrastructure for carrying out the study. All authors participated in writing and critically revising the manuscript and accepted the final version.

### PEER REVIEW

The peer review history for this article is available at https://publons.com/publon/10.1111/ejn.15882.

## Supporting information


**Figure S1.** Mean fALFF and ReHo maps of the neonate brain displayed on axial slices. Color bar denotes mean fALFF values. High mean fALFF values are located on the sensorimotor, parietal, temporal, visual, anterior prefrontal and basal ganglia regions. Mild asymmetry can be observed in temporal cortex and basal ganglia. Similarly high fALFF values in sensorimotor, visual and medial prefrontal regions have been previously reported in a larger sample (1).
**Figure S2.** Regions where fALFF significantly correlated with maternal age in years (p < 0.001 FWE‐corrected) in the naturally sleeping neonate (N = 21). Highlighted region entails the left superior frontal gyrus. Color bar represents T‐scores. Images are displayed in radiological convention on the UNC neonate template in axial, coronal and sagittal slices. Abbreviations: A = Anterior, P = Posterior, L = Left, R = Right.
**Figure S3.** Regions where fALFF significantly correlated with maternal composite score (p < 0.001 FWE‐corrected) in the naturally sleeping neonate (N = 21) with non‐parametric complementary model. Highlighted region entails the ventromedial prefrontal cortex. Color bar represents T‐scores. Images are displayed in radiological convention on the UNC neonate template in axial and sagittal slices. Abbreviations: A = Anterior, P = Posterior, L = Left, R = Right.
**Figure S4.** One‐sample T‐test results displaying mean connectivity from seed ROI (mPFC) in the neonate brain (N = 21). Horizontal rows correspond to connectivity patterns with distinct significance thresholds (with Z‐score 2.1 corresponding to p < 0.05; Z‐score 2.6 to p < 0.005; Z‐score 3.1 to p < 0.001; all multiple comparison corrected at cluster‐level). Seed ROI (x = 88, y = 148, z = 59 in the UNC template space) is illustrated on the right‐side brain image. Color bar represents Z‐scores. Images are displayed in radiological convention on the UNC neonate template. Abbreviations: mPFC = medial prefrontal cortex; ROI = region‐of‐interest, A = Anterior, P = Posterior, R = Right, L = Left.
**Figure S5.** Brain regions where maternal composite score was positively correlated (Z‐score threshold of 2.6, corresponding to p < 0.005 multiple comparison corrected at cluster‐level) with neonate mPFC seed connectivity in multiple regression analysis (N = 21). For mPFC seed ROI definition see supplementary materials, Figure 4. Images are displayed in radiological convention on the UNC neonate template. Abbrevations: mPFC = medial prefrontal cortex; ROI = region‐of‐interest, A = Anterior, P = Posterior, R = Right, L = Left.
**Figure S6.** Regions where fALFF significantly correlated with maternal SCL‐score (p < 0.001 FWE‐corrected) in the naturally sleeping neonate (N = 21). Highlighted region entails the ventromedial prefrontal cortex. Color bar represents T‐scores. Images are displayed in radiological convention on the UNC neonate template in axial and sagittal slices. Abbreviations: A = Anterior, P = Posterior, L = Left, R = Right.
**Figure S7.** Regions where fALFF significantly correlated with maternal EPDS‐score (p < 0.001 FDR‐corrected) in the naturally sleeping neonate (N = 21). Highlighted region entails the ventromedial prefrontal cortex. Color bar represents T‐scores. Images are displayed in radiological convention on the UNC neonate template in axial and sagittal slices. Abbreviations: A = Anterior, P = Posterior, L = Left, R = Right.
**Table S1.** Correlation matrix for metrics involved in this study. rs = Spearman rank correlation coefficient. * and ** denote statistically significant correlation at p < 0.05 and p < 0.01 levels, respectively.
**Table S2.** Model results with cluster coordinates, sizes and locations. Cluster coordinates are defined in the UNC neonate template (MNI).
**Table S3.** Whole sample estimated motion parameters and with correlations to metric Z‐scores (fALFF, SCA).

## Data Availability

The Finnish law and ethical permissions do not allow the sharing of the data used in this study.
