# Supplementary material for: Maternal psychological distress associates with alterations in resting‐state low‐frequency fluctuations and distal functional connectivity of the neonate medial prefrontal cortex
Source: Eur J Neurosci. 2022 Dec 17;57(2):242–57. doi: 10.1111/ejn.15882 (PMC10108202; doi:10.1111/ejn.15882)
Supplement: Supplementary file 1 — Figure S1. Mean fALFF and ReHo maps of the neonate brain displayed on axial slices. Color bar denotes mean fALFF values. High mean fALFF values are located on the sensorimotor, parietal, temporal, visual, anterior prefrontal and basal ganglia regions. Mild asymmetry can be observed in temporal cortex and basal ganglia. Similarly high fALFF values in sensorimotor, visual and medial prefrontal regions have been previously reported in a larger sample (1). Figure S2. Regions where fALFF significantly correlated with maternal age in years (p < 0.001 FWE‐corrected) in the naturally sleeping neonate (N = 21). Highlighted region entails the left superior frontal gyrus. Color bar represents T‐scores. Images are displayed in radiological convention on the UNC neonate template in axial, coronal and sagittal slices. Abbreviations: A = Anterior, P = Posterior, L = Left, R = Right. Figure S3. Regions where fALFF significantly correlated with maternal composite score (p < 0.001 FWE‐corrected) in the naturally sleeping neonate (N = 21) with non‐parametric complementary model. Highlighted region entails the ventromedial prefrontal cortex. Color bar represents T‐scores. Images are displayed in radiological convention on the UNC neonate template in axial and sagittal slices. Abbreviations: A = Anterior, P = Posterior, L = Left, R = Right. Figure S4. One‐sample T‐test results displaying mean connectivity from seed ROI (mPFC) in the neonate brain (N = 21). Horizontal rows correspond to connectivity patterns with distinct significance thresholds (with Z‐score 2.1 corresponding to p < 0.05; Z‐score 2.6 to p < 0.005; Z‐score 3.1 to p < 0.001; all multiple comparison corrected at cluster‐level). Seed ROI (x = 88, y = 148, z = 59 in the UNC template space) is illustrated on the right‐side brain image. Color bar represents Z‐scores. Images are displayed in radiological convention on the UNC neonate template. Abbreviations: mPFC = medial prefrontal cortex; ROI = region‐of‐interest, A = Anterio [file EJN-57-242-s001.pdf]

Supplementary material to

Maternal psychological distress associates with alterations in resting-state low-frequency fluctuations and distal functional connectivity of the neonate medial prefrontal cortex

Olli Rajasilta[1] MD, Suvi Häkkinen[1] PhD, Malin Björnsdotter[2,3] PhD, Noora M. Scheinin[1,4] MD, PhD, Satu J. Lehtola[1] MD, Jani Saunavaara[5] PhD, Riitta Parkkola[6] MD PhD, Tuire Lähdesmäki[7] MD PhD, Linnea Karlsson[1,8,9,11] MD PhD, Hasse Karlsson[1,4,8] MD PhD, Jetro J. Tuulari[1,4,9,10] MD, PhD.

1. FinnBrain Birth Cohort Study, Turku Brain and Mind Center, Institute of Clinical Medicine, University of Turku, Turku, Finland
2. The Sahlgrenska University Hospital, Gothenburg, Sweden
3. Department of Clinical Neuroscience, Karolinska Institutet, Stockholm, Sweden
4. Department of Psychiatry, University of Turku and Turku University Hospital, Turku, Finland
5. Department of Medical Physics, University of Turku and Turku University Hospital, Turku, Finland
6. Department of Radiology, University of Turku and Turku University Hospital, Turku, Finland
7. Department of Pediatric Neurology, Turku University Hospital and University of Turku, Finland
8. Center for Population Health Research, University of Turku and Turku University Hospital
9. Department of Psychiatry, University of Oxford, UK (Sigrid Juselius Fellowship)
10. Turku Collegium for Science and Medicine, University of Turku, Turku, Finland
11. Department of Paediatrics and Adolescent Medicine, University of Turku and Turku University Hospital, Turku, Finland

Corresponding author
Olli Rajasilta | operaj@utu.fi
FinnBrain Birth Cohort Study, Turku Brain and Mind Center
Lemminkäisenkatu 2, 20520, Turku, Finland

The purpose of this supplementary material is to provide the reader with:

1. Mean fALFF and ReHo maps of the neonate brain at 26.14 ± 6.28 days after birth
2. Effects of additional independent variables (IV) on primary models (sensitivity analyses):
    a. Neonate birth weight
    b. Maternal age
3. Exclusion/subgroup analysis without subjects with exposure to illicit substances/alcohol (N=18)
    a. Composite score model
4. Non-parametric composite score model results
5. Correlation matrix of measures used in this study
6. Cluster coordinates and effect sizes of parametric, non-parametric composite score and sensitivity analysis results
7. One sample T-test of mPFC seed connectivity
8. Group-level multiple regression results of PSE on neonate mPFC FC at a more stringent threshold
9. Estimated motion parameters table
10. Parametric fALFF results of SCL and EPDS score models

1. Mean fALFF and ReHo maps (N=21) of the neonate brain at 26.14 ± 6.28 days after birth.

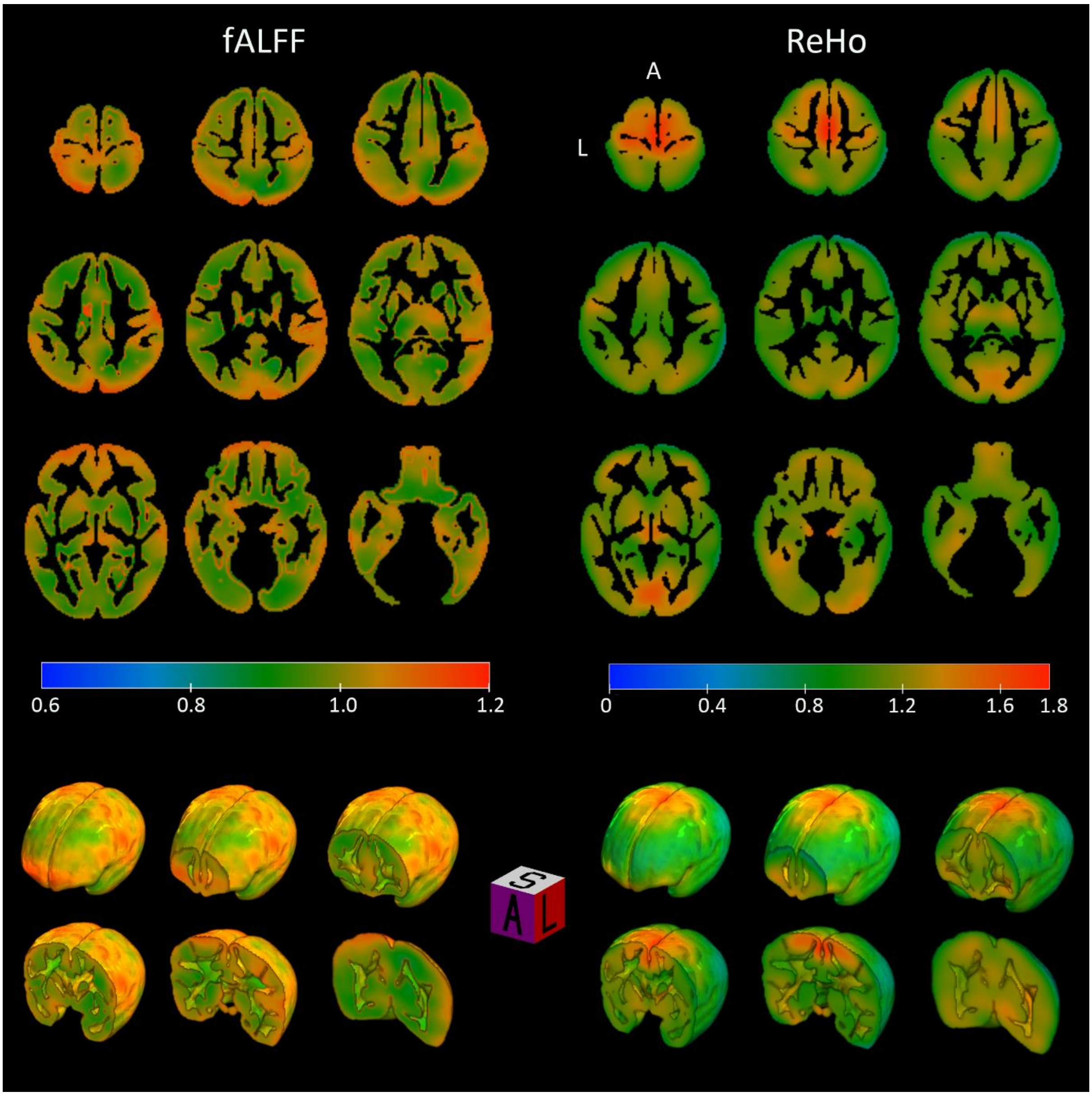

Supplementary materials, Figure 1. Mean fALFF and ReHo maps of the neonate brain displayed on axial slices. Color bar denotes mean fALFF values. High mean fALFF values are located on the sensorimotor, parietal, temporal, visual, anterior prefrontal and basal ganglia regions. Mild asymmetry can be observed in temporal cortex and basal ganglia. Similarly high fALFF values in sensorimotor, visual and medial prefrontal regions have been previously reported in a larger sample (1).

2. **Effects of additional independent variables on primary models (sensitivity analyses):**
    a. **Composite score and neonate birth weight**

In this multiple regression model, neonate birth weight was set as a 4th independent variable (IV) of no interest. Otherwise, we used prior default IVs: Neonate age at scanning (days), neonate sex and maternal pre-pregnancy BMI. The complete model thus consisted of the beforementioned IVs and composite score as the main explanatory variable (EV).

In this model, the effect observed in our main analysis of maternal composite score on neonate fALFF maps was reduced to statistical insignificance at $p < 0.001$ and $p < 0.005$ levels. No clusters that passed the multiple comparison statistical thresholding were detected.

Bivariate correlation analysis performed in SPSS revealed a significant correlation between neonate birth weight and composite score ($r_s = -0.606$). Variance of inflation (VIF) analysis revealed no indications of multicollinearity (VIF = 1.479). Even though there is an established link between neonate birth weight and exposure to prenatal stress (1), there is little reason to believe that infant birth weight itself would be the driving factor for effects seen in neonate fALFF maps. Nevertheless, to test this possibility we generated an additional model with infant birth weight as the EV and with neonate age at scanning, sex, and maternal pre-pregnancy BMI as the IVs. No statistically significant effects at $p < 0.001$ or $p < 0.005$ FWE-corrected level were obtained in this model.

We conclude that the mitigation of results in our main model, when corrected for neonate birth weight, was caused by the high negative correlation between neonate birth weight and composite score.

    b. **Composite score and maternal age in years**

Here, the multiple regression design consisted of four IVs of no interest: Neonate age at scanning (days), neonate sex, maternal pre-pregnancy BMI and maternal age in years. Composite score was set as the main EV.

The effects were reduced to statistical insignificance at $p < 0.001$ and $p < 0.005$ levels. The performed bivariate correlation analysis revealed that maternal age in years had a significant correlation with maternal pre-pregnancy BMI ($r_s = 0.570$), but no significant correlation was observed between composite score and maternal age. VIF analysis showed no indications of multicollinearity (VIF = 1.549) in this model.

Maternal age has been established to associate with levels of mental distress during pregnancy (2). To test whether maternal age had an independent effect on neonate fALFF maps, we performed another analysis with neonate age at scanning, neonate sex and maternal pre-pregnancy BMI as IVs. In this model, maternal age was set as the main EV. Here, we found a statistically significant (at $p < 0.001$ level) effect localizing to the left superior frontal gyrus ($p < 0.001$ FWE-corrected, cluster size of 903 voxels). The results are displayed in Supplementary materials figure 2.

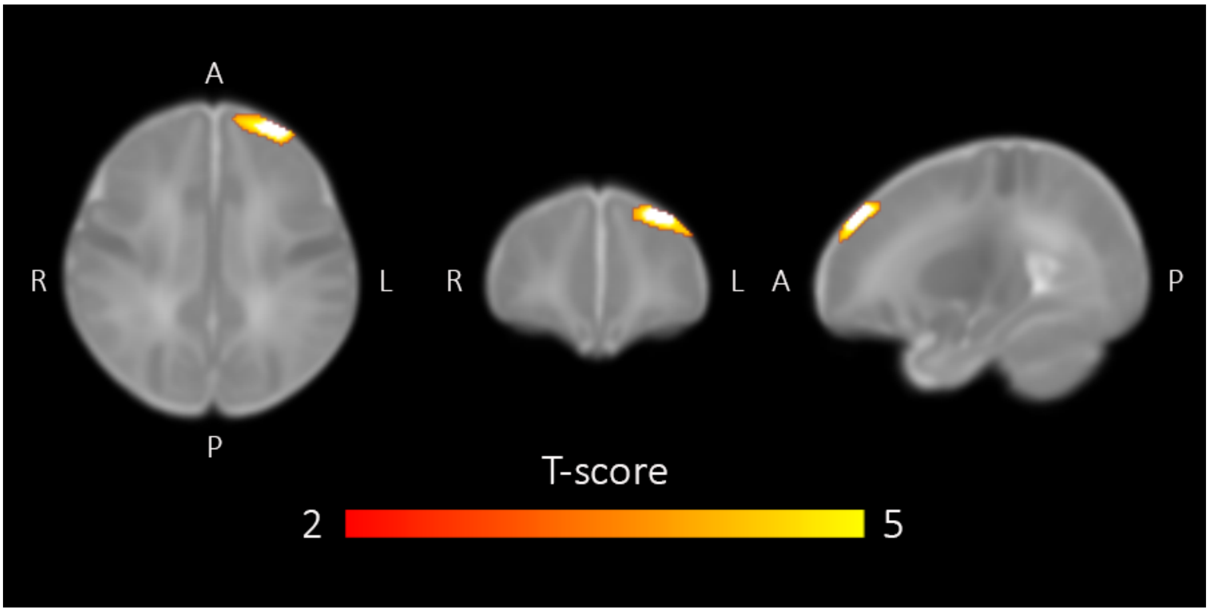

Supplementary materials, figure 2. Regions where fALFF significantly correlated with maternal age in years ($p < 0.001$ FWE-corrected) in the naturally sleeping neonate (N = 21). Highlighted region entails the left superior frontal gyrus. Color bar represents T-scores. Images are displayed in radiological convention on the UNC neonate template in axial, coronal and sagittal slices. Abbreviations: A = Anterior, P = Posterior, L = Left, R = Right.

Out of the five papers (3–7) investigating PSE effects on neonate rs-fMRI metrics, only one controlled for maternal age at beginning of pregnancy (4), with similar maternal age distribution as in our sample. They found no association between maternal age and neonate FC maps. Considering extant literature and the results of these sensitivity analyses, we cannot rule out that the effects of maternal psychological distress on offspring brain development might depend on maternal age.

3. Exclusion analysis (N=18)

To make sure the results of our main model were not influenced by exposure to illicit substances and/or alcohol, we performed an additional analysis, in which the exposed subjects were excluded.

a. Composite score model

In this model, we excluded the three subjects that were exposed to illicit substances (cannabis) and/or alcohol *in utero*, yielding a sample size of 18 subjects. Otherwise, identical design was used as in the main parametric model with neonate age at scanning, neonate sex and maternal pre-pregnancy BMI set as IVs. The composite score was set as the main EV. Statistical significance threshold was set to $p < 0.001$.

We obtained near-identical results with the exclusion analysis as with our main model with 21 subjects. A statistically significant effect was observed in the neonate mPFC ($p < 0.001$ uncorrected; $p < 0.001$ FWE-corrected; kE 796). Here, the cluster shape was slightly altered (supplementary materials, figure 2) and fragmented into two separate clusters.

Although minimally altered, this model yielded a highly comparable result to our main model with 21 subjects. In all cases, exposure to alcohol and/or illicit substances was mild. After consideration, we decided to include the three subjects exposed to alcohol for increased statistical power in our main model.

4. Non-parametric main model results (SnPM13)

To test the validity of the underlying assumptions in the main parametric model, we repeated the analysis using non-parametric permutation testing with the Statistical Non-Parametric Mapping software (SnPM13). As the parametric model, the non-parametric model had the following measures set as IVs: neonate age at scanning, neonate sex and maternal pre-pregnancy BMI. Composite score was set as the main EV. Statistical significance threshold was set to $p < 0.001$.

We found identical results as with the parametric model. The composite score – fALFF effect localized to the neonate mPFC ($p < 0.001$ uncorrected; $p < 0.001$ FWE-corrected; kE 794). No additional statistically significant clusters nor negative associations were observed.

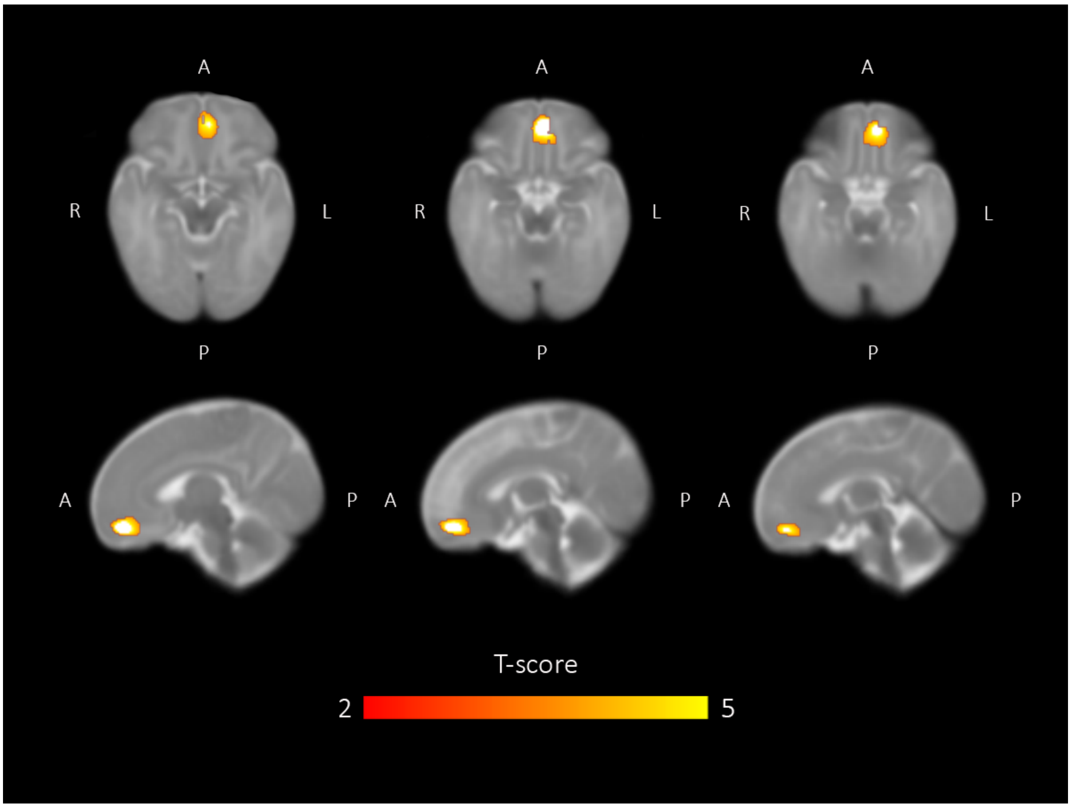

Supplementary materials, figure 3. Regions where fALFF significantly correlated with maternal composite score ($p < 0.001$ FWE-corrected) in the naturally sleeping neonate (N = 21) with non-parametric complementary model. Highlighted region entails the ventromedial prefrontal cortex. Color bar represents T-scores. Images are displayed in radiological convention on the UNC neonate template in axial and sagittal slices. Abbreviations: A = Anterior, P = Posterior, L = Left, R = Right.

5. **Supplementary materials, table 1.** Correlation matrix for metrics involved in this study. r_s = Spearman rank correlation coefficient. * and ** denote statistically significant correlation at $p < 0.05$ and $p < 0.01$ levels, respectively.

	Maternal age in years	Neonate birth weight	Maternal pre-pregnancy BMI	SCL-90	EPDS	Composite score
Maternal age in years	r_s = 1.000<br>p = N/A	r_s = -0.038<br>p = 0.871	r_s = 0.570**<br>p = 0.007	r_s = -0.193<br>p = 0.402	r_s = -0.112<br>p = 0.629	r_s = -0.126<br>p = 0.586
Neonate birth weight	r_s = -0.038<br>p = 0.871	r_s = 1.000<br>p = N/A	r_s = 0.200<br>p = 0.385	r_s = -0.447<br>p = 0.042	r_s = -0.675**<br>p = 0.001	r_s = -0.606**<br>p = 0.004
Maternal pre-pregnancy BMI	r_s = 0.570**<br>p = 0.007	r_s = 0.200<br>p = 0.385	r_s = 1.000<br>p = N/A	r_s = -0.500*<br>p = 0.021	r_s = -0.342<br>p = 0.129	r_s = -0.465*<br>p = 0.033
SCL-90	r_s = -0.193<br>p = 0.402	r_s = -0.447<br>p = 0.042	r_s = -0.500*<br>p = 0.021	r_s = 1.000<br>p = N/A	r_s = 0.599**<br>p = 0.004	r_s = 0.845**<br>p = 0.000
EPDS	r_s = -0.112<br>p = 0.629	r_s = -0.675**<br>p = 0.001	r_s = -0.342<br>p = 0.129	r_s = 0.599**<br>p = 0.004	r_s = 1.000<br>p = N/A	r_s = 0.911**<br>p = 0.000
Composite score	r_s = -0.126<br>p = 0.586	r_s = -0.606**<br>p = 0.004	r_s = -0.465*<br>p = 0.033	r_s = 0.845**<br>p = 0.000	r_s = 0.911**<br>p = 0.000	r_s = 1.000<br>p = N/A

6. **Supplementary materials, table 2.** Model results with cluster coordinates, sizes and locations. Cluster coordinates are defined in the UNC neonate template (MNI).

**Model**<br>(**EV** and IVs)	**p-threshold**	**p (FWE-corrected)**	**Cluster size in voxels (kE)**	**Cluster peak coordinates (X, Y, Z)**
• **Composite score**<br>• Neonate age at scanning<br>• Neonate sex<br>• Maternal pre-pregnancy BMI	p < 0.001	p < 0.001	794	2, 44, -29
**Non-parametric designs of the original model (SnPM13)**				
• **Composite score**<br>• Neonate age at scanning<br>• Neonate sex<br>• Maternal pre-pregnancy BMI	p < 0.001	p < 0.001	794	2, 44, -29
**Additional models (sensitivity analyses)**				
• **Composite score**<br>• Neonate age at scanning<br>• Neonate sex<br>• Maternal pre-pregnancy BMI<br>• Neonate birth weight	p < 0.01	p < 0.007	1601	2, 44, -29<br>3, 53, -19<br>10, 36, -30
• **Composite score**<br>• Neonate age at scanning<br>• Neonate sex<br>• Maternal pre-pregnancy BMI<br>• Maternal age	p < 0.05	p < 0.023	5418	1, 44, -29<br>1, 53, -22<br>3, 29, -31

7. One sample T-test of mPFC seed connectivity

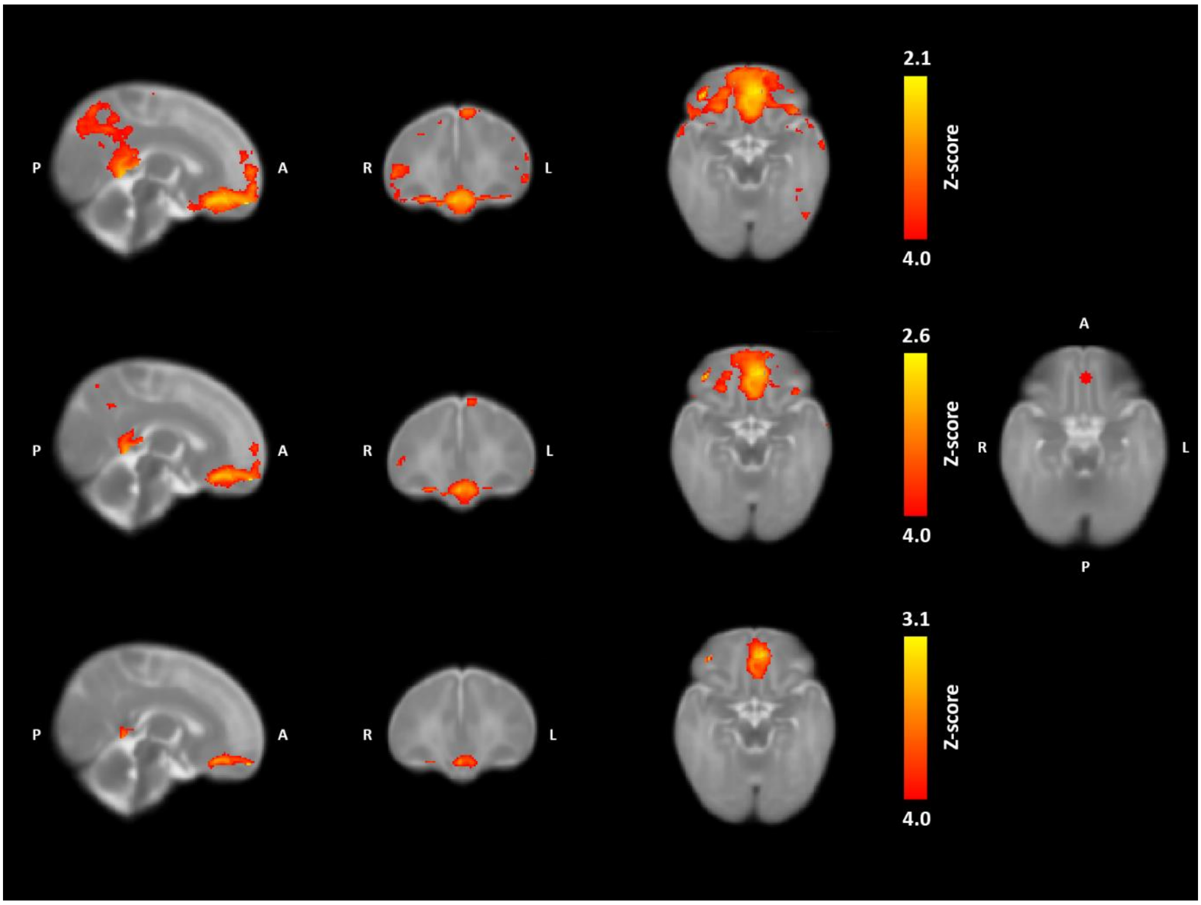

Supplementary materials, figure 4. One-sample T-test results displaying mean connectivity from seed ROI (mPFC) in the neonate brain (N = 21). Horizontal rows correspond to connectivity patterns with distinct significance thresholds (with Z-score 2.1 corresponding to $p < 0.05$; Z-score 2.6 to $p < 0.005$; Z-score 3.1 to $p < 0.001$; all multiple comparison corrected at cluster-level). Seed ROI (x = 88, y = 148, z = 59 in the UNC template space) is illustrated on the right-side brain image. Color bar represents Z-scores. Images are displayed in radiological convention on the UNC neonate template. Abbreviations: mPFC = medial prefrontal cortex; ROI = region-of-interest, A = Anterior, P = Posterior, R = Right, L = Left.

8. Group-level multiple regression results of PSE on neonate mPFC FC at more stringent threshold.

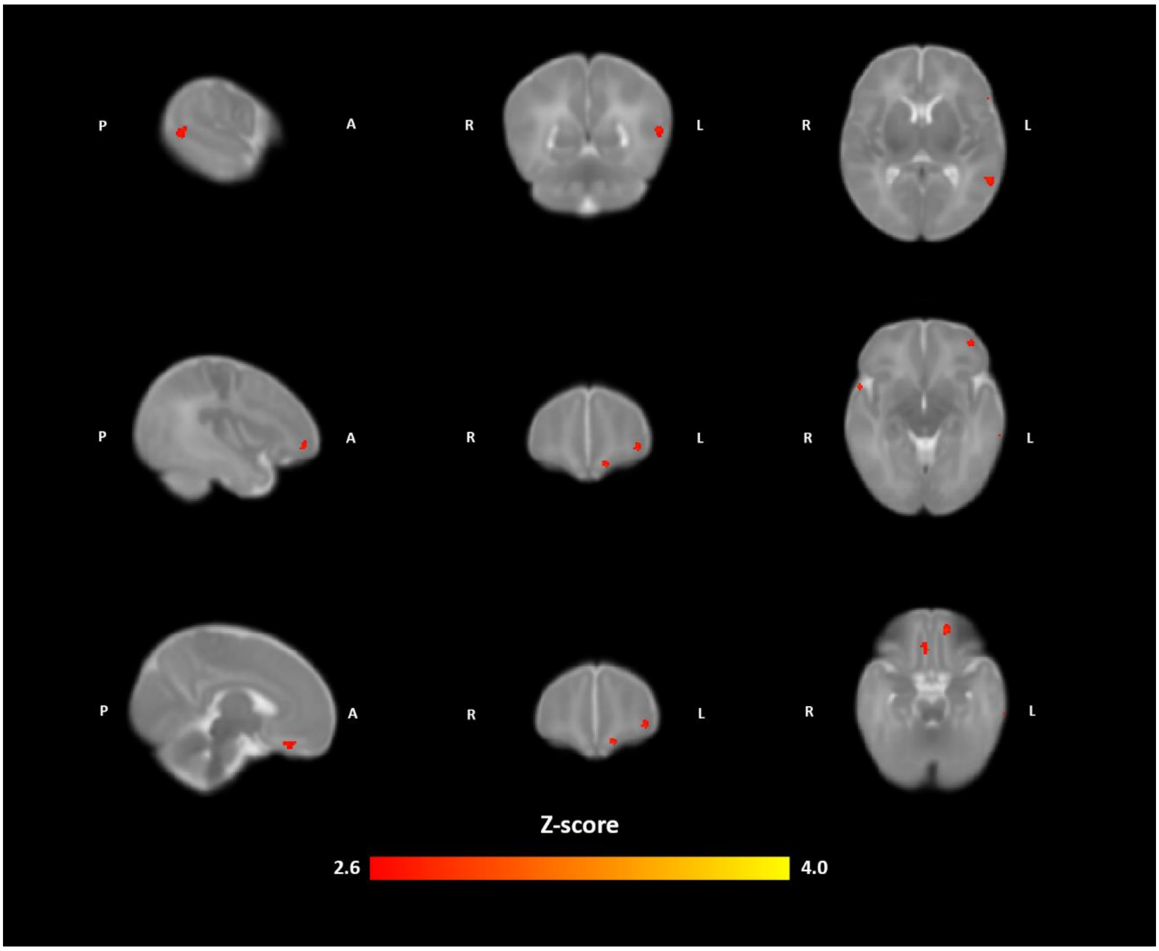

Supplementary materials, figure 5. Brain regions where maternal composite score was positively correlated (Z-score threshold of 2.6, corresponding to $p < 0.005$ multiple comparison corrected at cluster-level) with neonate mPFC seed connectivity in multiple regression analysis (N = 21). For mPFC seed ROI definition see supplementary materials, figure 4. Images are displayed in radiological convention on the UNC neonate template. Abbrevations: mPFC = medial prefrontal cortex; ROI = region-of-interest, A = Anterior, P = Posterior, R = Right, L = Left.

9. Estimated motion parameters.

Variable	Absolute mean	Max	Min	fALFF, Z-score r_s (p-value)	SCA, Z-score r_s (p-value)
Composite motion estimate (mm)	0.53	1.97	0.05	0.192 (0.378)	-0.242 (0.291)
DVARS	1,16	10,58	0,87	0.243 (0.332)	0.185 (0.463)
Translation (X) (rad)	0.13	0.46	-0.12	-0.023 (0.920)	-0.035 (0.880)
Translation (Y) (rad)	0.27	0.07	-2.80	-0.234 (0.308)	-0.292 (0.199)
Translation (Z) (rad)	0.55	0.54	-3.13	-0.039 (0.867)	0.165 (0.475)
Rotation (X) (rad)	0.02	0.07	-0.17	0.073 (0.754)	-0.075 (0.746)
Rotation (Y) (rad)	0.01	0.09	-0.06	-0.326 (0.149)	0.121 (0.602)
Rotation (Z) (rad)	0.04	6.49	-0.00	0.000 (1.000)	-0.142 (0.540)

Supplementary materials, table 3. Whole sample estimated motion parameters and with correlations to metric Z-scores (fALFF, SCA).

10. Parametric fALFF results of SCL and EPDS score models

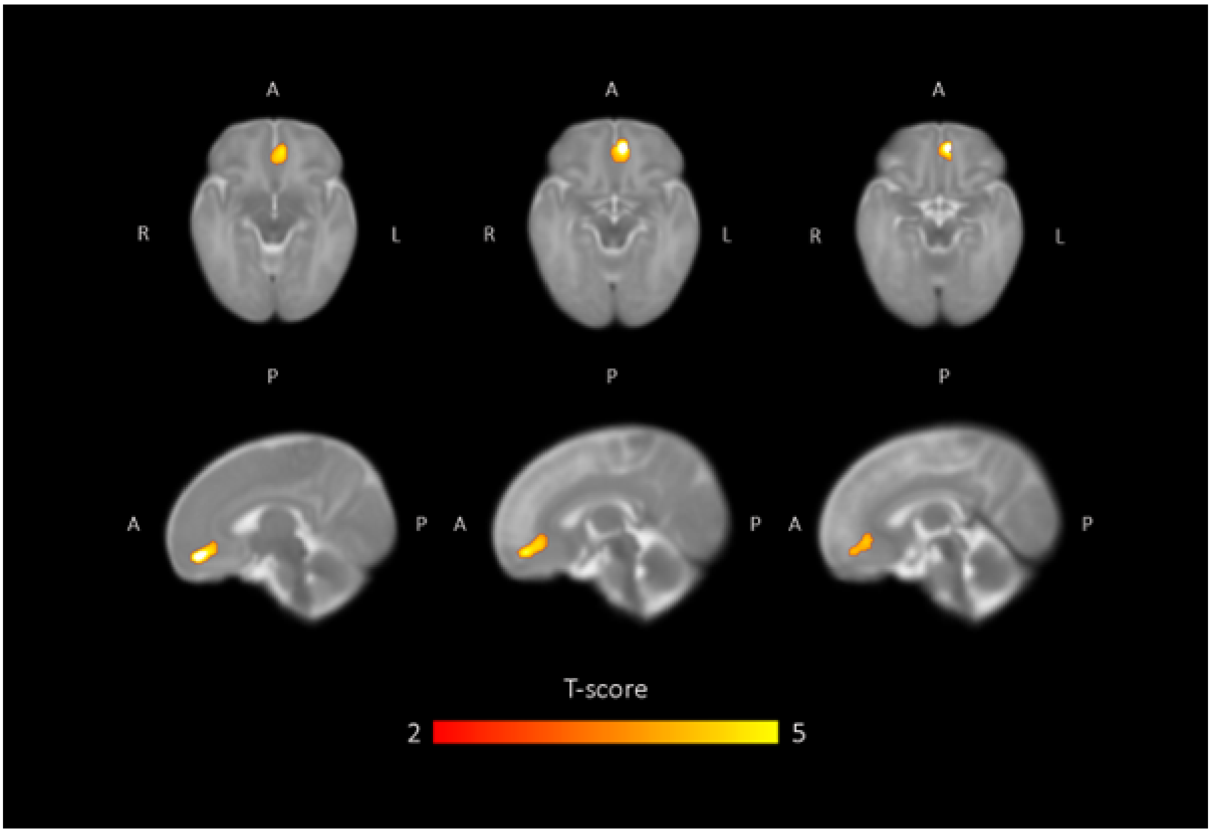

Supplementary materials, figure 6. Regions where fALFF significantly correlated with maternal SCL-score ($p < 0.001$ FWE-corrected) in the naturally sleeping neonate ($N = 21$). Highlighted region entails the ventromedial prefrontal cortex. Color bar represents T-scores. Images are displayed in radiological convention on the UNC neonate template in axial and sagittal slices. Abbreviations: A = Anterior, P = Posterior, L = Left, R = Right.

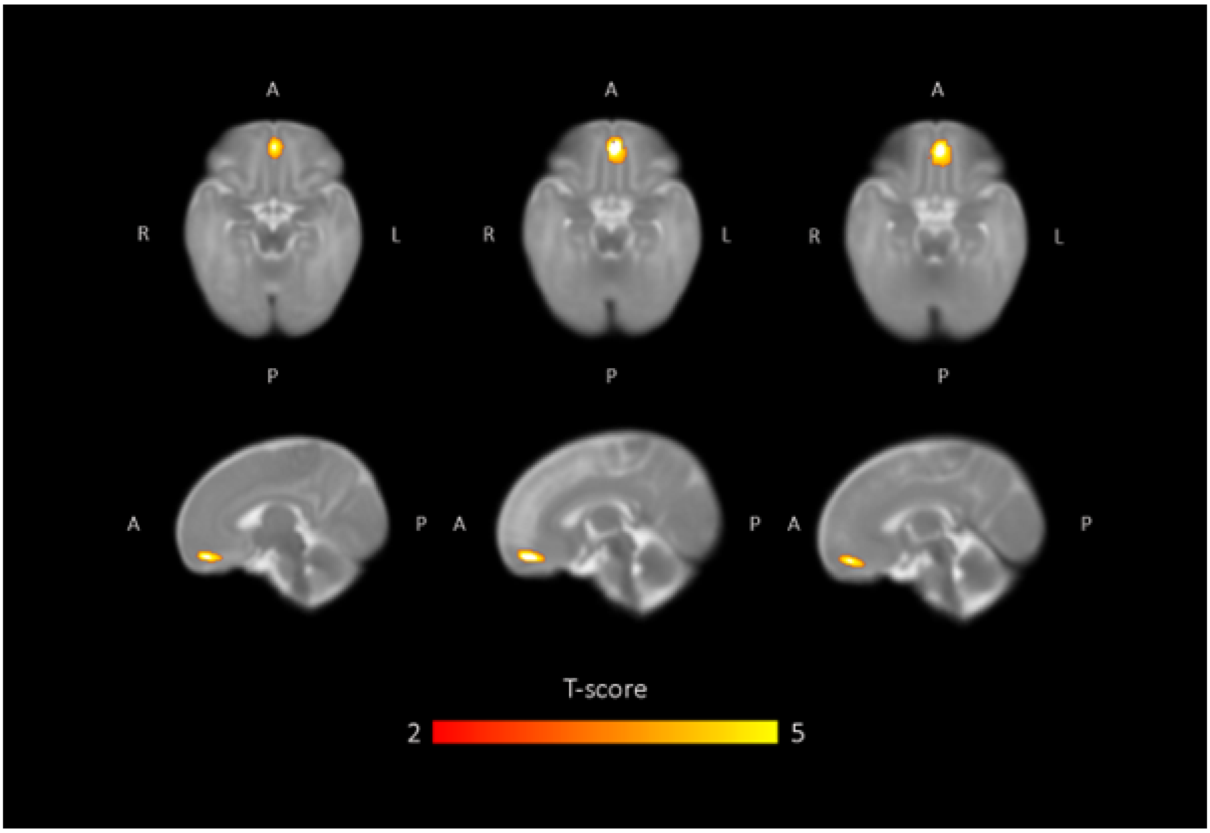

Supplementary materials, figure 7. Regions where fALFF significantly correlated with maternal EPDS-score ($p < 0.001$ FDR-corrected) in the naturally sleeping neonate ($N = 21$). Highlighted region entails the ventromedial prefrontal cortex. Color bar represents T-scores. Images are displayed in radiological convention on the UNC neonate template in axial and sagittal slices. Abbreviations: A = Anterior, P = Posterior, L = Left, R = Right.

REFERENCES

1. Huang Z, Wang Q, Zhou S, Tang C, Yi F, Nie J. Exploring functional brain activity in neonates: A resting-state fMRI study. Dev Cogn Neurosci. 2020;45.

2. Aasheim V, Waldenström U, Hjelmstedt A, Rasmussen S, Pettersson H, Schytt E. Associations between advanced maternal age and psychological distress in primiparous women, from early pregnancy to 18 months postpartum. BJOG An Int J Obstet Gynaecol. 2012;119(9):1108–16.

3. Qiu A, Anh TT, Li Y, Chen H, Rifkin-Graboi A, Broekman BFP, et al. Prenatal maternal depression alters amygdala functional connectivity in 6-month-old infants. Transl Psychiatry. 2015;5(2).

4. Posner J, Cha J, Roy AK, Peterson BS, Bansal R, Gustafsson HC, et al. Alterations in amygdala-prefrontal circuits in infants exposed to prenatal maternal depression. Transl Psychiatry. 2016;6(11):e935-8.

5. Scheinost D, Kwon SH, Lacadie C, Sze G, Sinha R, Constable RT, et al. Prenatal stress alters amygdala functional connectivity in preterm neonates. NeuroImage Clin. 2016;12:381–8.

6. Spann MN, Monk C, Scheinost D, Peterson BS. Maternal immune activation during the third trimester is associated with neonatal functional connectivity of the salience network and fetal to toddler behavior. J Neurosci. 2018;38(11):2877–86.

7. Scheinost D, Spann MN, McDonough L, Peterson BS, Monk C. Associations between different dimensions of prenatal distress, neonatal hippocampal connectivity, and infant memory. Neuropsychopharmacology [Internet]. 2020;45(8):1272–9. Available from: http://dx.doi.org/10.1038/s41386-020-0677-0
